# Cytoplasmic convection currents and intracellular temperature gradients

**DOI:** 10.1371/journal.pcbi.1007372

**Published:** 2019-11-04

**Authors:** Rachel Howard, Aaron Scheiner, Jessica Cunningham, Robert Gatenby

**Affiliations:** Department of Integrated Mathematical Oncology, Moffitt Cancer Center, Tampa, FL, United States of America; Oxford, UNITED KINGDOM

## Abstract

Intracellular thermometry has recently demonstrated temperatures in the nucleus, mitochondria, and centrosome to be significantly higher than those of the cytoplasm and cell membrane. This local thermogenesis and the resulting temperature gradient could facilitate the development of persistent, self-organizing convection currents in the cytoplasm of large eukaryotes. Using 3-dimensional computational simulations of intracellular fluid motion, we quantify the convective velocities that could result from the temperature differences observed experimentally. Based on these velocities, we identify the conditions necessary for this temperature-driven bulk flow to dominate over random thermal diffusive motion at the scale of a single eukaryotic cell. With temperature gradients of the order 1°C and diffusion coefficients comparable to those described in the literature, Péclet numbers ≥ 1 are feasible and permit comparable or greater effects of convection than diffusion in determining intracellular mass flux. In addition to the temperature gradient, the resulting flow patterns would also depend on the spatial localization of the heat source, the shape of the cell membrane, and the complex intracellular structure including the cytoskeleton. While this intracellular convection would be highly context-dependent, in certain settings, convective motion could provide a previously unrecognized mechanism for directed, bulk transport within eukaryotic cells.

## Author summary

Individual cells are the building blocks of all living things, and a myriad of processes occur within their walls. To facilitate the generation of energy and manufacturing of proteins and other molecules critical for cell survival, work and growth, intracellular material often must be shuttled to different locations or compartments within the cell. It is vital that we understand this intracellular transport if we hope to fully understand cell functioning. Some mechanisms of intracellular transport are well established, including random diffusive motion and active transport by molecular motor proteins. Recently, experimental evidence has suggested there are areas of substantial local heating within the cell, introducing the possibility of temperature gradient-driven convective circulation. Here, we explore the theoretical possibility of such mechanisms competing with diffusion to drive intracellular flows, and identify the conditions necessary for this to occur. For intracellular material with low diffusivity and in the presence of temperature gradients described in the recent literature, simulations suggest this mode of intracellular transport may indeed feasible and should be further validated experimentally.

## Introduction

The cytoplasm of a cell is a hive of activity, facilitating many processes crucial for cell functioning. While protein biosynthesis and glycolysis, transport of metabolites, and signal transduction from the cell membrane to the nucleus or organelles can occur in the cytosol [1–5], many critical molecules (including nutrients and proteins synthesized in the ribosomes) need to be shuttled to different locations or compartments within the cell to perform their respective functions [6]. Thus, a detailed understanding of all influences on intracellular fluid motion and substance transport must necessarily precede a complete understanding of cell functioning.

Thermal diffusive motion can produce a characteristic mean squared displacement of small molecules in the cytoplasm. This is typically assumed to be the primary source of substance transport in prokaryotic cells lacking organelles and compartmentalization [2,7]. In eukaryotic cells, cytoskeletal tracks (actin, kinesins, microtubules) provide direct pathways for the active transport of relatively small packets of intracellular cargo via transport vesicles and molecular motor proteins [3,7]. Directional flow of cytosol has also been widely observed in the cells of plants, fungi and, more recently, animal and human cells [8–12]. This cytosolic motion, or cytoplasmic streaming, is characterized by a circulatory pattern (cyclosis) [10], yet the mechanisms driving this circulation are still a topic of debate [10,13–16]. Regardless of mechanism, complex flow patterns in the cytoplasm are of critical importance due to their ability to redistribute materials throughout the cell and enhance nutrient transport and overall cell functioning [16].

Temperature strongly influences biochemical reactions, gene expression, membrane ion transport, protein-protein interactions and overall cell functioning [17–23]. While intracellular temperature is notoriously difficult to measure, recent advances in thermometry have allowed high-resolution temperature mapping within plant and animal cells [24,25]. Results suggest both the nucleus and specific organelles can act as local heat sources, leading to localized variations in intracellular temperature [26–30]. Such variations depend on biochemical reaction processes; excess energy can be transformed into heat and released exothermically [29]. In the nucleus, DNA replication, transcription, and RNA processing could all be contributing to local thermogenesis [31]. In the mitochondria, local heat generation is likely a result of active energy metabolism, with a potential contribution from proton transfer through the mitochondrial inner membrane [28,32,33]. The potential for mitosis, hydrolysis of ATP/GTP or phosphorylation of centrosomal proteins to induce local temperature increases at the centrosome has also been discussed [34,35], and calcium pump activity may also cause local heating in the endoplasmic reticulum [36]. Cell cycle changes in the temperature difference between the nucleus and the cytoplasm have also been observed, and are believed to be attributable to fluctuations in the temperature of the cytoplasm caused by the cell-cycle dependent activity of ribosomal synthesis and corresponding increased biochemical activity [26].

The recently described local heat sources and proximal temperature gradients could have significant implications for intracellular fluid motion and transport. In the presence of temperature differences across the cell (e.g. between the nucleus or perinuclear mitochondria and the cooler cell membrane) thermal instability could result in self-organizing convective currents, as observed in many other natural settings [37–39]. These cytoplasmic currents could contribute to the movement of macromolecules and organelles, substance transport, cytosolic mixing and ultimately cell functioning. The possibility of intracellular buoyancy-driven convective transport in large cells was first addressed over three decades ago [40], yet in the absence of accurate measurements of intracellular temperature and *in vivo* cytoplasmic velocities, the feasibility of buoyancy-driven convective motion dominating over thermal motion and fluid viscosity to drive intracellular flow has not been thoroughly evaluated.

Here, we conduct three-dimensional computational simulations of cytosolic fluid motion within biologically realistic scale cellular domains sufficiently large to experience the conventional effects of gravity. We implement local temperature gradients that lie within the range of those recently observed experimentally [26–30], allowing visualization of the resulting flow field and estimation of the velocities characterizing the convective motion. Based on the results, we evaluate the theoretical feasibility of this temperature-induced convective circulation dominating over random diffusive motion at the scale of the single cell, and describe the conditions necessary for this to occur. If validated experimentally, cytoplasmic convection could be an important but previously unrecognized mechanism for intracellular transport in certain biological settings.

## Model

### Computational domain

**Fig 1:**
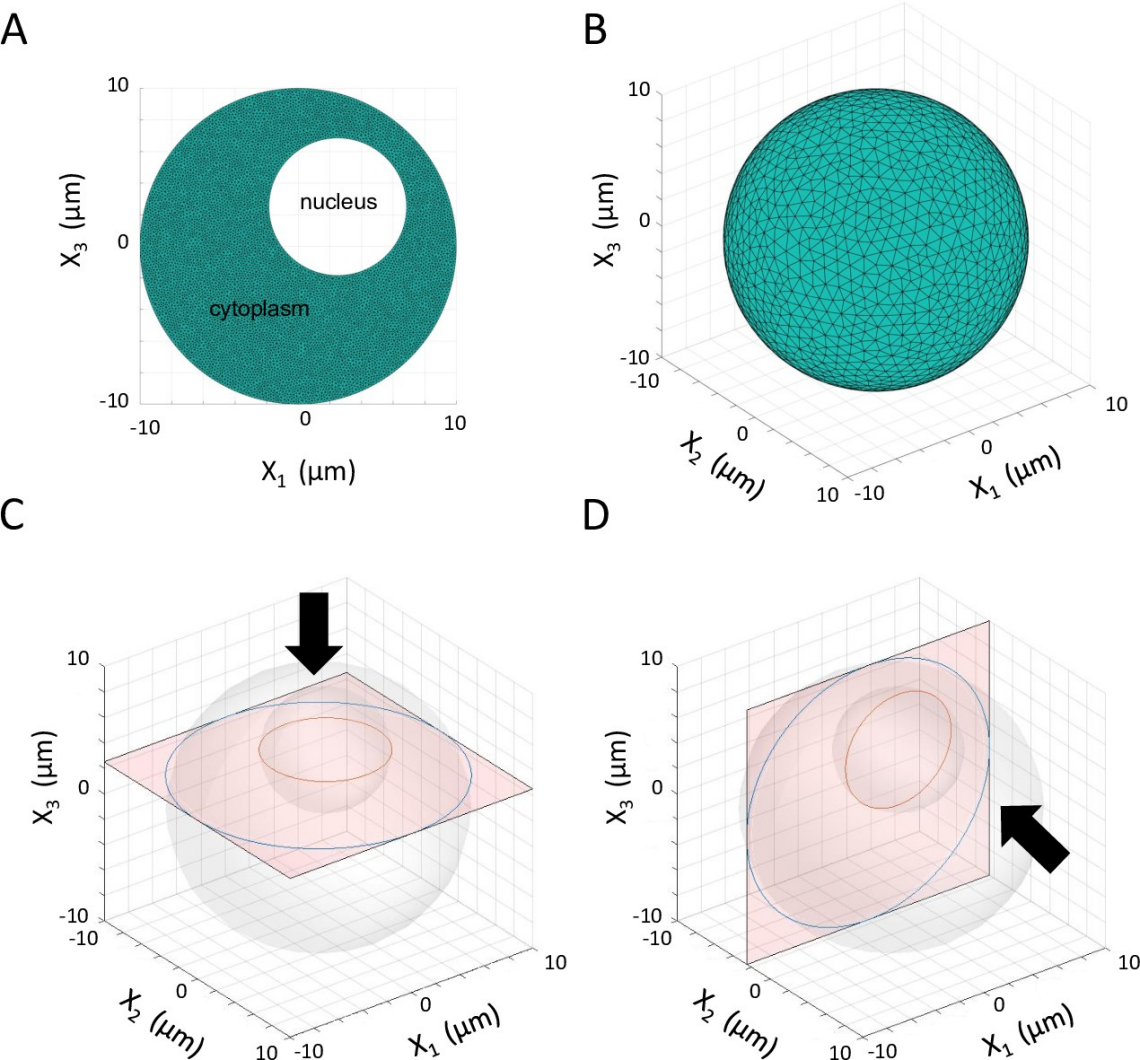
Two- and three-dimensional cell domains and computational grids. **A**. Two-dimensional domain and grid featuring 8,640 unique element nodes for spherical cell. **B**. Three-dimensional domain and grid featuring 10,324 nodes for spherical cell. Triangular grids optimized using Gmsh **C** Horizontal plane surface (x_1_-x_2_) used for demonstration of three-dimensional velocity flow fields. **D** Vertical plane surface (x_1_-x_3_) used for demonstration of three-dimensional velocity flow fields. All planes pass through midpoint of cell nucleus. Large arrows demonstrate the direction from which 3D results (presented on respective plane surface) will be viewed.

The computational domain is designed to be a simple representation of a spherical cell with off-center nucleus (**Fig 1A and 1B**). The radius was arbitrarily chosen to be 10μm, with a nuclear radius of 4.3μm defined such that the (three dimensional) volume of the nucleus is approximately 8% of the total cellular volume, motivated by recent literature [41]. The nucleus is defined as a hollow volume with a solid wall (no-slip) boundary. Diffusive processes through this boundary are not incorporated in the present model. The cell membrane represented by the outer domain boundary is also defined as a no-slip solid wall. The internal cytoskeletal structure and corresponding motor-driven active transport are excluded for the purposes of this exploratory study. Simulations were first conducted on a two-dimensional domain to aid description of the resulting flow field. For clarity, all three-dimensional results are presented on two-dimensional plane surfaces centered at the midpoint of the cell nucleus (**Fig 1C and 1D**). All computational domains and corresponding meshes described in this manuscript were generated using three-dimensional finite element mesh generator Gmsh [42].

#### Governing equations

The governing steady-state mass and momentum conservation equations are given by

$$\rho (u∙\nabla )u=-\nabla p+\mu \nabla^{2}u+\frac{\mu }{3}\nabla (\nabla ∙u)–\rho g\hat{z}$$

∇∙(ρu)=0

$$\nabla ∙(uT)=\kappa \nabla^{2}T$$

where, velocity ***u*** = [*u*_1_, *u*_2_, *u*_3_] (m s^-1^), pressure *p* (Pa) and temperature *T* (K) are the variables for which we wish to solve [43]. Velocity components *u*_1_, *u*_2_, *u*_3_ represent the velocity in a three-dimensional Cartesian coordinate system (x_1,_ x_2_, x_3_). As mentioned, the outer boundary (cell membrane, w_1_), and inner boundary (nucleus, w_2_) are treated as solid walls such that $u_{i}^{w_{1}}=u_{i}^{w_{2}}=0$, and fixed temperatures are imposed here ($T^{w_{2}}=310.15K;T^{w_{1}}\in [310.15K,323.15K]$). Relevant parameters are υ (m^2^ s^-1^) the kinematic viscosity, ρ (kg m^-3^) the fluid density, *g* (m s^-2^) gravity, and κ (m^2^ s^-1^) the thermal diffusivity. Values of all parameters are provided in **Table 1.** The density and temperature are assumed to be related via an equation of state *ρ* = *ρ*_0_(1−*β*(*T*−*T*_0_)), where β is the coefficient of thermal expansion. The following non-dimensionalization can be applied

$$x=L\bar{x}\;t=\frac{L}{U}\bar{t}\;u=U\bar{u}\;\rho =\rho_{0}\bar{\rho }\;p=\rho_{0}gL\bar{p}\;T=T_{0}+\theta \bar{T}$$

where an overbar denotes a dimensionless variable, L is the respective length scale, *ρ*_0_ and *T*_0_ are reference state static terms, *θ* is a typical small deviation from the reference temperature, and *U* is defined as $U=\frac{\rho_{0}gL^{3}}{\mu }$. This non-dimensionalization, after dropping the overbar, results in the following system of non-dimensional equations

$$Ga(1-\beta \theta T)(u∙\nabla )u=-\nabla p+\nabla^{2}u+\frac{1}{3}\nabla (\nabla ∙u)-(1-\beta \theta T)\hat{z}$$

∇∙((1−βθT)u)=0

$$Ra(u∙\nabla )T=\nabla^{2}T$$

where $Ga=\frac{gL^{3}}{\nu^{2}}$ and $Ra=\frac{UL}{\kappa }$ are the Galilei and Rayleigh numbers, respectively, and the equation of state becomes *ρ* = 1−*βθT*. Note that the above system of equations could be simplified further; the product *βθ*, and thus variations in density, are small, suggesting these terms can be considered negligible unless multiplied by the acceleration due to gravity [44]. Furthermore, as will be discussed in the Results, the Galilei and Rayleigh number may also be negligibly small based on the parameter values described in Table 1. Despite the potential for these simplifications, no terms were excluded in the computational solver.

**Table 1:** Computational model parameters assuming a base temperature of cytosolic fluid of approximately 37°C.

Parameter	Notation	Value/Range	SI unit
Kinematic viscosity	*υ*	0.6959 x10^-6^	m^2^ s^-1^
Gravity	g	9.81	m s^-2^
Thermal diffusivity	κ	λ/(ρCp)	m^2^ s^-1^
Thermal expansion coefficient	β	0.361 x10^-3^	K^-1^
Specific heat	C_p_	4.178	kJ (kgK)^-1^
Thermal conductivity	λ	0.6252	W (mK)^-1^
Density	ρ	993.38	kg m^-3^
Diffusion coefficient	D	1x10^-14^ to 1x10^-10^	m^2^ s^-1^

#### Numerical solution

The governing equations are solved using QuickerSim (QuickerSim Ltd, Warsaw, Poland), a finite element-based computational fluid dynamics toolbox for resolving fluid flows in the MATLAB environment (MathWorks Inc., Natick, MA). The finite element method (FEM) has been discussed in detail elsewhere [44]. To resolve the pressure-velocity coupling problem inherent in incompressible flows, the toolbox implements the semi-implicit method for pressure-linked equations (SIMPLE) [45]. The streamline upwind/Petrov-Galerkin (SUPG) stabilization method [46] is incorporated in the computational solver to avoid numerical instability in the form of spurious oscillations in the solution. Simulations were run until reaching a steady state. A maximum residual value of 1E-6 was imposed to ensure solution convergence. The complete toolbox used for all simulations in this manuscript and detailed tutorials on modeling natural fluid phenomena including convection are available at http://quickersim.com.

### Parameter selection

While various organelles and particles are suspended within the cytosol, around 70% of the cellular volume is water [47], leading us to assume certain fluid properties relating to water where equivalent values for eukaryote cytoplasm are not readily available. Hence, we use the thermal expansion coefficient, thermal conductivity and specific heat capacity of water as an estimate of the bulk properties of the cytosolic fluid. The viscosity of cytoplasm is also believed to be approximately equal to that of pure water [48], although diffusion through the cytoplasm is speculated to be slower. Diffusion coefficients of small particles have previously been found to range from the order of 1 *μm*^2^/*s* to as high as 150 *μm*^2^/*s* [49,50]. Several sources have suggested that large particles in the cytoplasm can exhibit diffusion rates well below this range, closer to 0.001 *μm*^2^/*s* [10,47]. In fact, in [10] it is suggested that for larger vesicles in the cytoplasm, “movement will be insignificant in the absence of active transport mechanisms” due to the particularly low diffusion rates observed here. Sub-diffusive behavior has also been observed in the cytoplasm [51,52], resulting in a below-linear increase in the mean squared displacement of particles over time. We thus consider a range of 0.01 to 100 *μm*^2^/*s* to be a fairly conservative estimate of the realistic range of diffusion coefficients in eukaryote cytoplasm, particularly as we do not wish to restrict our consideration of this transport mechanism to only the smallest proteins.

All parameters are listed in **Table 1**. Where necessary, these parameters are chosen to relate to the human body temperature of 37°C. Thermodynamic properties of water at a range of temperatures are widely documented in the literature; a concise summary can be found in [53]. Note that temperature will be converted to degrees Celsius for presentation of results. The kinematic viscosity is assumed constant for the purposes of this study.

We initiated all simulations with a neutral (37°C) temperature profile everywhere but the nuclear wall. A range of temperatures are imposed here, with particular emphasis on the range 37.001°C to 38°C based on recent observations of a ~1°C temperature differential between the heated nucleus and the outer cell membrane [25,26,54]. Larger local temperatures (up to 50°C in the mitochondria) have been reported in the literature [30]. While values of this magnitude should be further verified experimentally, we include local temperature differences up to this recently proposed 13°C for completeness. The maximum flow velocity will be identified for all simulations.

The phenomena that govern fluid flow at large scales are significantly different to those acting at the micro scale. Surface forces become increasingly important as volumes shrink, the relative effect of the gravity force is reduced (although note that at scales on the order of tens of micrometers such as the ones in the present manuscript we can still assume the gravity force is non-negligible [55]), and low Reynolds numbers typically result in laminar, creeping flows [56]. Based on the velocities obtained from the three-dimensional simulations and the parameters in Table 1, we will evaluate our theoretical flow fields by means of non-dimensional scaling parameters including the Reynolds, Galilei, Rayleigh and Péclet numbers, with particular emphasis on the ability of convective velocities of this magnitude to dominate over thermal diffusion.

### Complex domain examples

After validating the physical feasibility of convection-driven flow on the scale of a single cell, we consider several more complex hypothetical cellular domains. We investigate the influence of various biological properties including, but not limited to, the cell shape, nucleus size and location, the orientation of the cell, the size of the temperature gradient and the distribution of the heat source (for example the uneven spatial distribution of mitochondria around the nucleus) on the convective flow fields. This provokes discussion of the implications of convective currents on intracellular mixing and transport in more heterogeneous and biologically realistic cellular domains.

## Results

### Simulations

**Fig 2:**
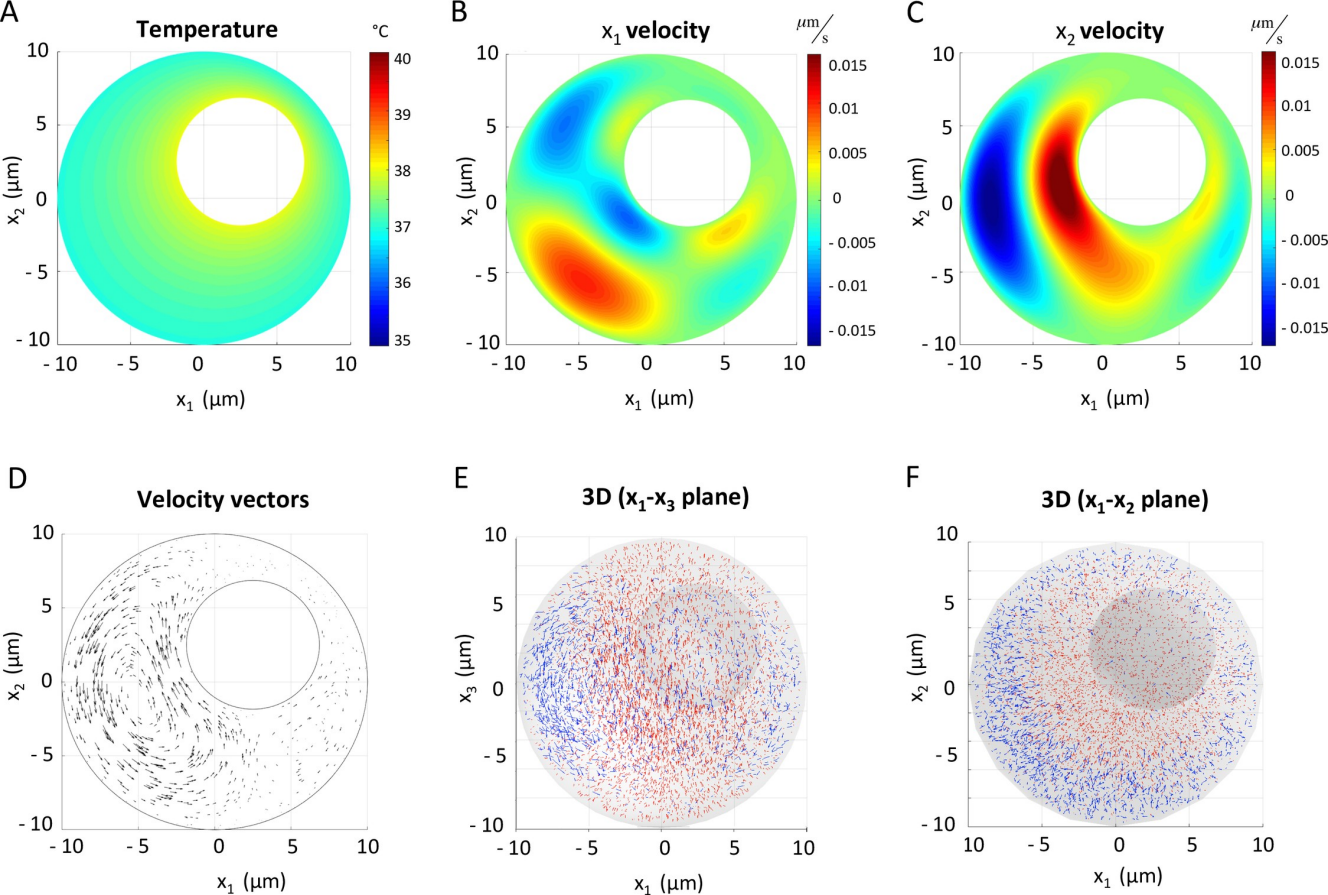
Simulation results for two- and three-dimensional spherical cell domains. **A.** Temperature profile across two-dimensional domain, with heated nuclear wall (38°C) and neutral cell membrane (37°C). **B,C**. Two-dimensional convection-induced horizontal (**B**; x_1_) and vertical (**C**; x_2_) velocity profiles demonstrating one major (left of nucleus) and one minor (right of nucleus) convective circulation structure characterized by central upwelling (red) and fountain-like downwelling (blue) around the outer cell wall. **D**. 2D velocity vectors with arrow length indicative of speed relative to maximum speed in domain. **E,F.** Equivalent velocity vectors on both vertical (**E**) and horizontal (**F**) planes in three-dimensional simulations. For clarity, vectors with a positive vertical (x_3_) component are shown in red, while those with a negative vertical component are shown in blue. The fountain-like structure of convective circulations is again clearly evidenced by large regions of upwelling in the center of the domain and the corresponding downwelling around the outer cell wall.

Preliminary simulations were conducted on the two-dimensional domain with a temperature of 38°C imposed at the nucleus wall, 1°C higher than that of the cytoplasm and cell membrane (**Fig 2**). Simulations were run until a steady state was reached. **Fig 2A** demonstrates the imposed temperature profile within the two-dimensional domain, with a constant temperature imposed at the nucleus. **Fig 2B and 2C** show the horizontal (x_1_) and vertical (x_3_) velocity profiles in the two-dimensional simulation. In **Fig 2C**, a region of strong upwelling (red; positive vertical velocity) can be observed in the center of the domain, around the central heated nucleus. Corresponding regions of downwelling (blue; negative vertical velocity) can be seen towards the exterior cell walls. Note that the domain setup (with off-center nucleus) dictates the distribution of up- and downwelling on either side of the cell; should the nucleus be perfectly centered, the flow would be symmetrical about a vertical axis centered in x_1._ To maintain a closed system, these regions of up- and down-welling are connected by corresponding negative and positive horizontal velocity regions forming complete closed circulations (**Fig 2C**). For the remainder of the manuscript these will be referred to as convective circulations or currents, avoiding the conventional term “convective cell” to avoid confusion with the biological cellular domain in question.

Intuitively, this pattern of motion corresponds to warm, less dense fluid rising around the heated nucleus, being horizontally displaced as it reaches the upper cell boundary, then cooling and sinking to create a “fountain” effect that redistributes fluid throughout the domain. **Fig 2D** demonstrates this closed convective circulation using non-dimensional velocity vectors, whose lengths are proportional to the maximum velocity observed in the whole domain such that longer arrows represent faster flow. Comparable simulations were conducted on a three dimensional domain to verify the convective structures. The fountain-like structure of the two-dimensional convective circulations is conserved in the three-dimensional case (**Fig 2E**). In **Fig 2E and 2F**, velocity vectors with a positive vertical (x_3_) component are shown in red, while those with a negative vertical component are shown in blue. Large regions of upwelling can be observed in the center of the domain on both vertical (**E**) and horizontal (**F**) planes, transporting fluid from the base to the top of the cell, past the heated cell nucleus. Again, when this warm fluid reaches the cell membrane, it is pushed outward and subsequently begins its descent, during which cooling occurs. Comparable results in a columnar cell domain can be found in **S1 Fig**. In **S2 Fig**, we additionally demonstrate concentration fields for an arbitrary species within this cellular domain at a temperature difference of 1°C and a range of diffusivities D, where concentration C satisfies

$$\nabla ∙(uC)=D\nabla^{2}C$$

**Fig 3:**
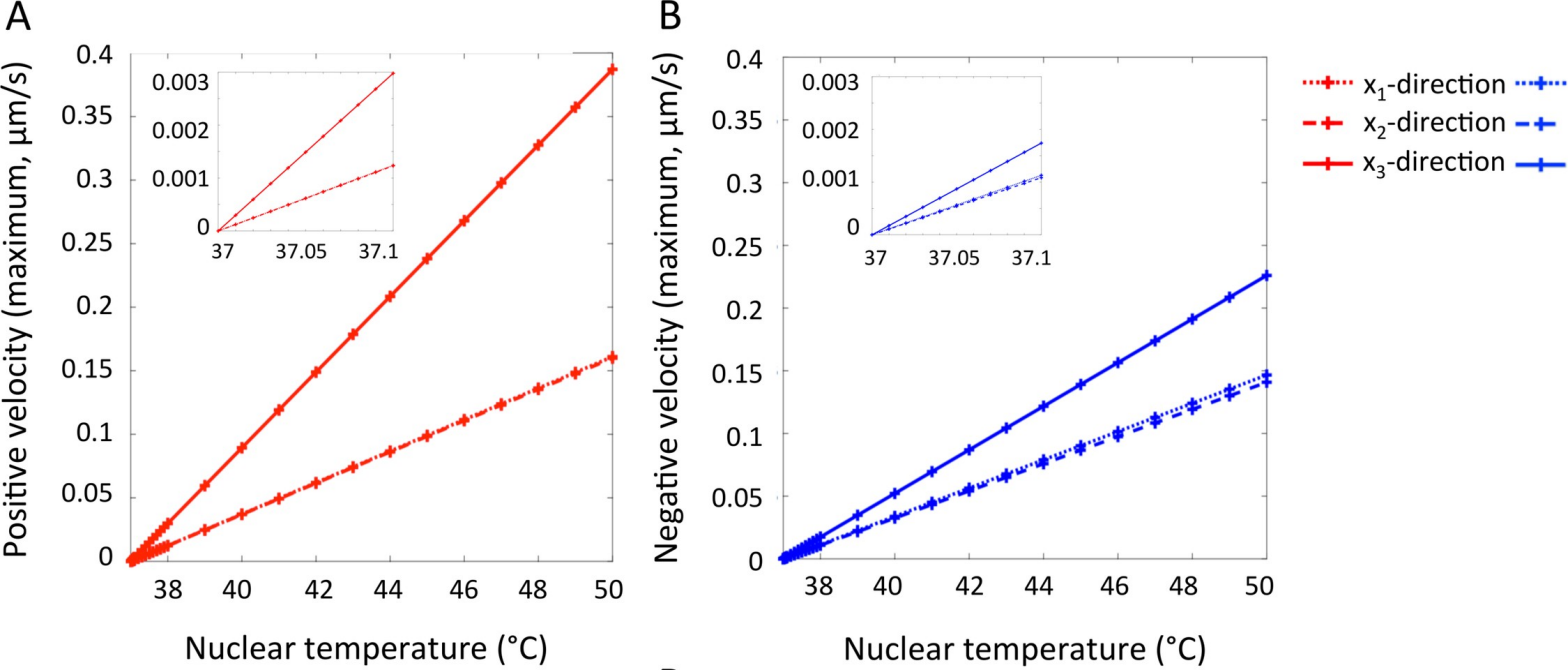
Maximum upwelling (**A**) and downwelling (**B**) velocities in each respective direction from three-dimensional simulations with a range of hypothetical nuclear temperatures ranging from 37°C to 50°C. Intuitively, a higher temperature gradient between the local heat source and the outer cell membrane induced the largest convective velocities, reaching approximately 0.39 μm/s in the positive vertical direction (**A**).

The nature of convective motion is well established. A more important question in the present setting is what rate of cytoplasmic flow could potentially be induced by convective motion on the spatial scales of interest to cell biologists, and what temperature gradients would be required for their occurrence? In addition to the above example (with only a 1°C temperature difference between the nucleus or perinuclear mitochondria and the cell membrane), simulations were also run with a wide range of hypothetical nuclear temperatures ranging from 37°C to 50°C. Intuitively, when the nuclear temperature is equal to the outer cell membrane temperature (37°C), the flow remains stationary. **Fig 3A and 3B** show the model-predicted flow velocities in the positive vertical (upwelling, **A**) and negative vertical (downwelling, **B**) directions induced by our range of temperature gradients. With a nuclear temperature of only 37.1°C, maximum upwelling and downwelling velocities can reach 0.003 μm/s and 0.002 μm/s, respectively. At 38°C these velocities become 0.03 μm/s and 0.02 μm/s. Should a local nuclear temperature of 50°C be biologically feasible, flow speeds up to 0.39 μm/s could be possible. Note that upwelling regions in other natural settings including ocean and atmospheric circulation also demonstrate higher velocities in the positive vertical direction, resulting from increased fluid temperatures and typically accompanied by a narrowing of the upwelling convective “limb” to ensure balancing of the respective upward and downward volume fluxes.

### Dimensional analysis & scaling

Based on the parameter values in Table 1, the reference length of the domain described in the current manuscript (L), and an arbitrary reference velocity within the range identified in our simulations (u^*^), the Reynolds ($Re=\frac{u^{*}L}{\nu }$) and Galilei ($Ga=\frac{gL^{3}}{\nu^{2}}$) numbers capturing the balance between inertial or gravitational forces and viscous forces are both <<1 in all simulated flows, and hence laminar flow typical of the Stokes flow regime is expected. Due to the small physical scale of the problem, the Rayleigh number ($Ra=\frac{UL}{\kappa }$) is also <<1. However, as we consider fluid between two spheres (concentric or eccentric) at different temperatures, convective flow will necessarily occur [57–59]. The important question becomes do velocities of this magnitude allow advection to compete with diffusion, and thus could this convective flow potentially aid cellular transport, metabolism, and overall cell functioning. For this to occur, we typically require a Péclet number ($Pe=\frac{u^{*}L}{D}$) of O(1). As an example, for a length scale of 10 μm and a flow rate of 0.01 μm/s, substances with a diffusion constant lower than 0.1 μm^2^/s will start to be affected by internal circulation. Given the range of diffusion coefficients described in the literature, particularly for large particles, this suggests that active transport by convection may be meaningful even in small cells.

**Fig 4:**
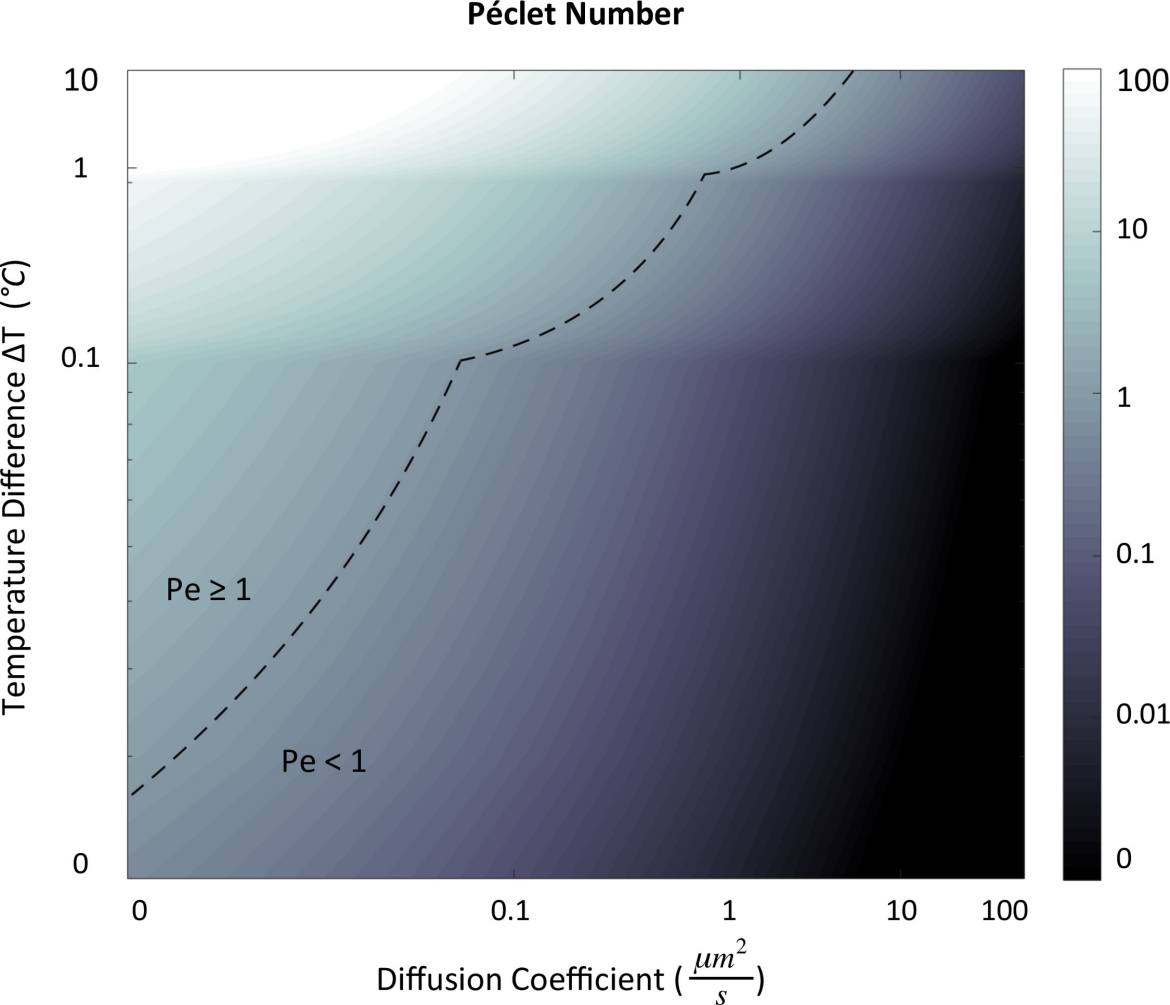
Péclet number (u*L/*D*) as a function of the temperature difference ΔT (Tmax−T_min_,°C) and the diffusion coefficient D (*μm*^2^/*s*), demonstrating regions in which Pe>1 and thus convection can theoretically dominate over diffusion.

**Fig 4** demonstrates the Péclet number (u*L/*D*) as a function of the temperature difference ΔT (Tmax−T_min_,°C) and the diffusion coefficient D (*μm*^2^/*s*). A length scale *L* of 20*μm* was used in the calculations for consistency with the diameter of the spherical cell shown throughout the manuscript. The velocities u^*^ used in the calculation of Pe were obtained from the results of a subset of 30 simulations with an imposed nuclear temperature ranging from 37.01°C to 47°C (ΔT ∈ (0.01, 10)).

**Fig 4** demonstrates that Péclet numbers of O(1) are feasible for many unique combinations of diffusion coefficient and temperature gradient that lie within the ranges described in the literature. A temperature gradient of only 1°C could theoretically permit convection-driven motion for particles experiencing diffusion coefficients approaching 1, orders of magnitude larger than some of the diffusion coefficients that have been described in existing studies. Note that if we were to consider a larger eukaryote such that length scale L was >20*μm*, Péclet numbers of O(1) would be expected in an even larger proportion of the parameter combinations considered. Similarly, if diffusion coefficients of 0.001 *μm*^2^/*s* are indeed possible, the number of parameter sets meeting this criteria would increase further still. In contrast, it is also important to note that for molecules with diffusion coefficients above 100 *μm*^2^/*s*, including ATP, Fig 4 suggests convective motion is likely have minimal to no impact on intracellular motion.

Given that diffusion may still influence flow patterns to some extent even when the Péclet number is around or greater than 1, we also plot individual particle trajectories at a range of diffusion coefficients and temperature gradients to evaluate the respective contributions of each transport mechanism (**S3 Fig**). These results suggest a slightly lower diffusion coefficient or higher temperature gradient may be required than the Peclet number alone would imply, but support the possibility of convection-driven flow at values described in the literature cited in this manuscript.

### Complex domain examples

**Fig 5:**
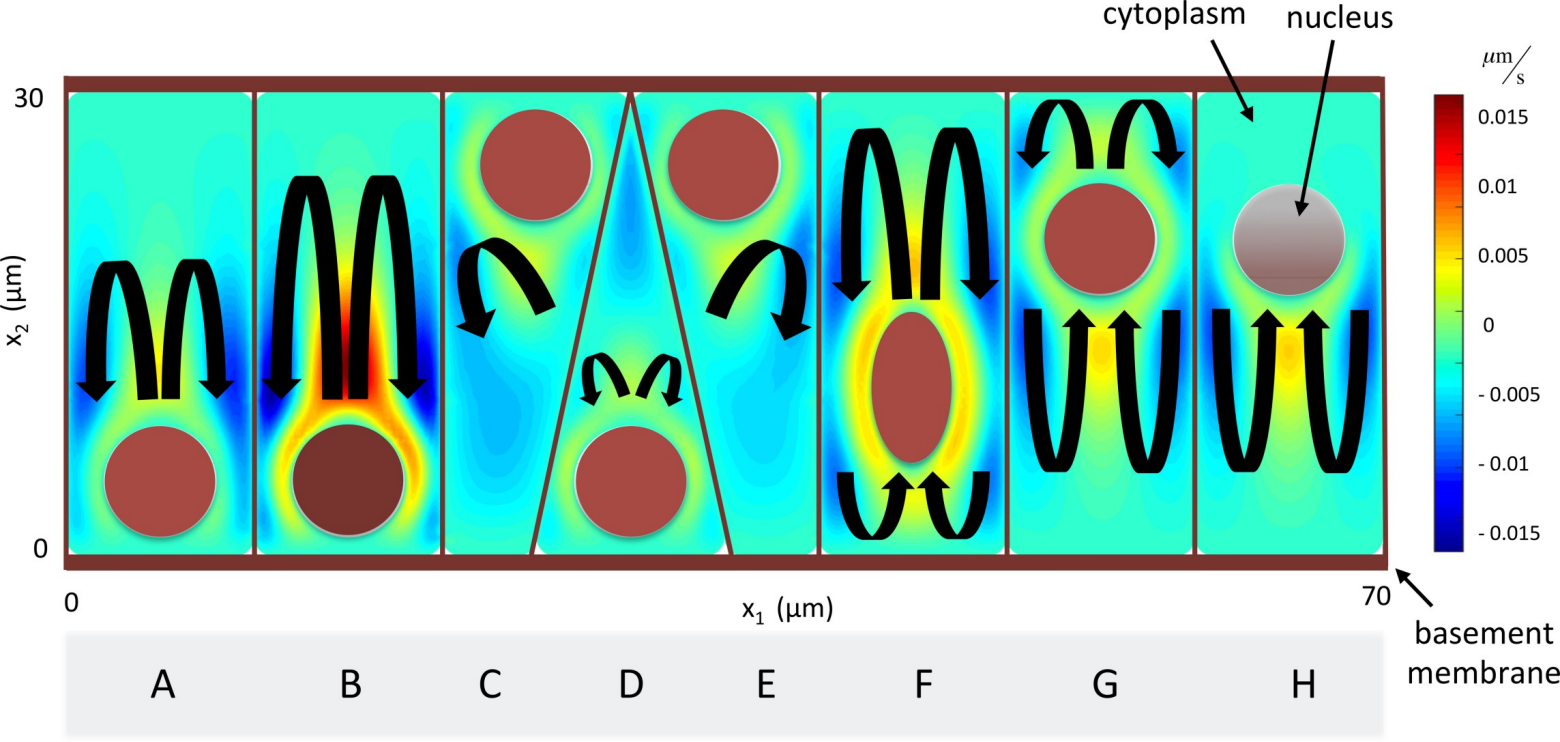
Simulated flow patterns within an 8-cell domain representative of a series of columnar epithelial cells, demonstrating the influence of: (**A**) increased nuclear heating (**B**), pseudo-stratification (**C-E**), altered shape of nucleus (**F**), altered location of nucleus (**G**) and uneven distribution of heating (**H**). Overlaid schematic provides a conceptual representation of the nuclear shape and temperature (brown ellipses), and the flow direction (black arrows).

Should convection-driven cytoplasmic flow be feasible, the flow patterns observed and their respective velocities would depend on many factors including, but not limited to, the nucleus shape, size and location, the orientation of the cell, the size of the temperature gradient, and the distribution of the heat source (for example the uneven spatial distribution of mitochondria around the nucleus). Further simulations on a more complex model domain are presented in **Fig 5**, demonstrating the potential influence of some of these factors on a simple flow field in columnar and pseudo-stratified epithelial cells. Several observations can be made: increasing the nuclear temperature can increase the proportion of the cytoplasm being entrained in the flow (**B**), the location of the nucleus and distribution of heat source can have a significant effect on flow patterns (**G,H**), and certain cell structures (**B,F**) generate the most uniform flow distribution throughout the domain.

**Fig 6:**
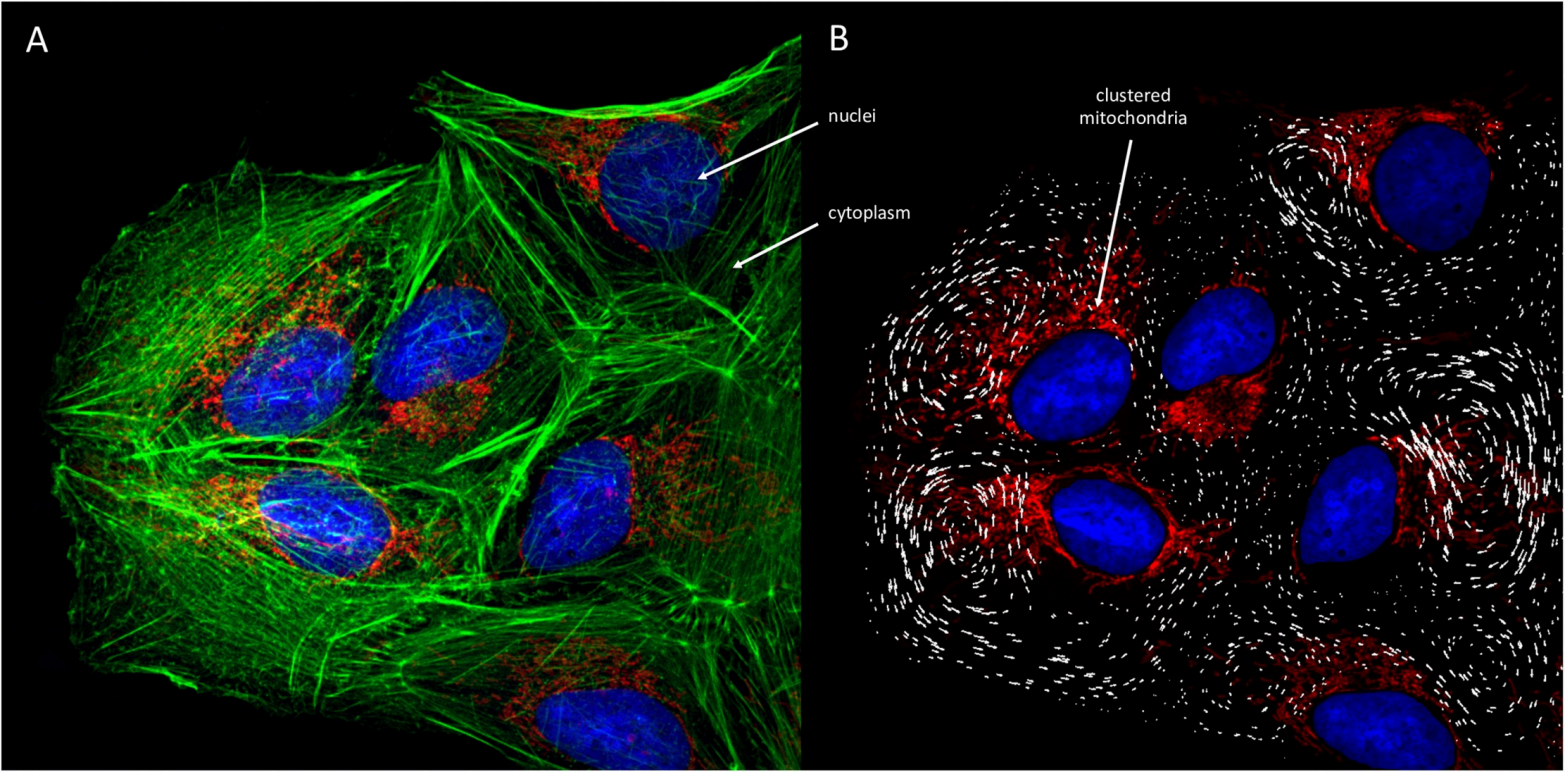
**A**. Immunofluorescence images of Bovine Pulmonary Artery Endothelial Cells (purchased from ThermoFisher Scientific) to provide a biologically realistic domain for application of the present model. Nuclei shown in blue are stained using DAPI, mitochondria in red (note clustering around cell nuclei) are stained using MitoTracker Red CMKRos, and cytoskeleton in green stained with Alexa Fluor 488 Phalloidin. **B**. Distribution of cell nuclei, mitochondria and the shape of the cell membranes (based on the distribution of the microtubules) extracted from the fluorescence image and used to develop a unique representative computational grid for each cell. Simulations are conducted with heating applied only in regions of mitochondrial clustering. The resulting flow field in terms of velocity vectors is overlaid on the original image, demonstrating the circulatory patterns hypothetically generated in this multi-cell domain. Note that the convective flows will typically be both within the x_1_-x_2_ plane of the image and also in the perpendicular x_3_ direction. These models assume no inter-cellular flow. However, cyclosis could promote movement of fluid, ions, and small molecules through gap junctions.

As a further example, we analyzed a six-cell cluster of bovine pulmonary artery endothelial cells in which histological stains permitted identification of the distribution of mitochondria around the nucleus, position of the nucleus within the cell, the size of the cell, and the shape of the cell membrane. By assuming a fixed orientation and imposing local heating only in the regions of mitochondrial clustering, as opposed to imposing a fixed temperature at the nuclear wall as in our earlier simulations, we generated model-predicted cyclosis fields on this experimental domain (**Fig 6**). Note that this still serves as a simplified model problem: the microtubules and microfilaments of the cytoskeleton would likely compartmentalize tor otherwise influence the flow field and this will be the subject of future investigations. We also assumed that no flow occurred between the cells. However, if contiguous cells were connected through gap junctions, the intracellular cyclosis could significantly affect this mechanism of cell-cell communication.

## Discussion

In living cells, diffusion and directed transport by molecular motor proteins are thought to be the primary drivers of intracellular fluid motion. Here, we test the hypothesis that temperature gradient-induced convective flow could play a significant role in intracellular movement of ions, molecules and organelles in certain conditions, generating intracellular mixing and serving as a complementary mechanism of transport to those already established. We consider example geometries with the approximate scale and fluid parameters of eukaryotic cells, and impose intracellular temperature gradients on the order of those observed experimentally. Results suggest that while convective effects in excess of diffusive effects may be uncommon, and only applicable to certain classes of molecule, in the setting of large cells they may indeed be feasible. We further consider individual particle trajectories to evaluate how flow patterns may vary as the relative contribution of convection and diffusion varies. While this provides additional validation that under certain conditions convection may drive particle motion, the required temperature gradients are slightly higher than our analysis of the Péclet number alone would suggest. Additionally, flow patterns and rates would likely depend on cell size and shape, the location of the nucleus, and the temperature and distribution of the mitochondria or other local heat source.

While there are many biochemical reactions within the nucleus, mitochondria and other organelles that could be resulting in local heat production, determining the maximum temperature change that could potentially be caused *in vivo* by these mechanisms is a difficult task. To date, such assessments have been primarily theoretical, and have led to much debate in the published literature [25,26,60]. A recent conclusion has been that in order to fully understand thermal dynamics within cells, intracellular heat sources and their thermal interaction with intracellular molecules should be chemically assessed to develop a theory for mesoscopic non-equilibrium thermodynamics [25]. To date, this has not been achieved. Ultimately, heat transfer at micro- and nano-scales is still not particularly well described by conventional thermodynamics, and the feasibility of these temperature variations needs to be verified in future work. Despite the controversy surrounding the theoretical underpinnings of intracellular temperature gradients, we believe the existing experimental evidence of significant temperature differences between the cytoplasm and both the nucleus and the mitochondria (25, 30, 53) is compelling, and sufficient to warrant theoretical investigations into the implications of such temperature gradients for intracellular transport and function.

Several simplifications were made during model design. The current model omits cytoskeletal intricacies including membranous subcompartments and issues relating to cytosolic macromolecular crowding, which are likely to influence the observed cytosolic flow patterns. Actomyosin is also inherently contractile, and the resultant forces could also influence the flow field and correspondingly the magnitude of effect of local heating-induced convection. Extension of the present work to more complex model domains including these various cytoskeletal features is a promising area for further study. Furthermore, we primarily focus on the ability of convection to dominate over diffusion, concluding that these mechanisms likely both contribute to intracellular mixing with the respective contribution of each mechanism being context-dependent. Inclusion of intracellular transport via molecular motor proteins in future simulations could also permit further demonstration of how these different transport mechanisms operate in synergy. Diffusion into and out of the nucleus and the outer cell wall are also not accounted for; the description of solid no-slip walls with fixed temperature boundary conditions is clearly a simplification of a much more complex biological system. The orientation of the cell relative to gravity would of course also influence the flow patterns described herein.

While convection has not traditionally been considered an important driver of intracellular fluid motion, in light of recent observations of significant intracellular temperature gradients, a more thorough evaluation of this mechanism is necessitated. Any additional understanding of the mechanisms of intracellular motion could have wide reaching implications, beyond the realm of basic cell biology to disease and therapy. For example, given that mitochondrial clustering is associated with both increased nuclear reactive oxygen species (ROS) and the ATP-dependent de novo protein synthesis known to occur in rapidly dividing cells [61,62], such clustering may prove to be more common in cancer cells than previously appreciated. The recently suggested high temperature of mitochondria coupled with the observation of cyclosis-like patterns of cytoplasmic flow in human cancer cells suggests further investigation into the potential evolutionary benefits of temperature-induced cytoplasmic motion.

## Supporting information

S1 Fig Simulation results for two- and three-dimensional columnar cell cases. **A.** Temperature profile across two-dimensional domain, with heated nuclear wall (38°C) and neutral cell membrane (37°C). **B,C**. Two-dimensional convection-induced horizontal (**B**; x_1_) and vertical (**C**; x_2_) velocity profiles, again demonstrating one major (left of nucleus) and one minor (right of nucleus) convective circulation structure characterized by central upwelling (red) and fountain-like downwelling (blue) around the outer cell wall. **D**. 2D velocity vectors with arrow length indicative of speed relative to maximum speed in domain. **E,F.** Equivalent velocity vectors on both vertical (**E**) and horizontal (**F**) planes in three-dimensional simulations. Again, vectors with a positive vertical (x_3_) component are shown in red, while those with a negative vertical component are shown in blue.
(PDF)

S2 Fig Concentration fields for an arbitrary substance with diffusivity D ranging from 0.00001 *μm^2^/s* to 10 *μm^2^/s*. In column A, the flow of this arbitrary substance is from outside the cell (high concentration imposed at outer cell wall), and in column B, the substance is originating from the nucleus (high concentration imposed at nuclear wall). At a diffusivity of 0.1 *μm^2^/s*, the concentration field begins to be influenced by convection. At a diffusivity of 0.001 *μm^2^/s*, the concentration field is clearly aligned with the flow velocity profile. At low diffusivities (0.00001 *μm^2^/s*, it can be seen that material originating at the cell membrane, and material originating at the nucleus, tend to cluster on opposite sides of the cell as a result of the offset nucleus and uneven distribution of the “convective cell” structure.
(PDF)

S3 Fig Particle tracking results. **A.** Zoomed-in view of start point for all simulated particle trajectories ([-2,-2], indicated by white star). Red arrows indicate that the bulk convection-driven flow is in the direction of the upper left corner of the panel. **B**. Individual particle trajectories (from start point again indicated by white star) for a particle of density 1050 *kg/m*^*3*^ and diameter 0.1 *μm* in flows governed by diffusion coefficients ranging from order 0.1 to order 0.00001 and temperature gradients ranging from 1°C to 13°C. Diffusion coefficients < O(0.001) or temperature gradients > 3°C are required for convection-dominated flow with this specific set of parameter values.
(PDF)

## References

[1] Berg JM, Tymoczko JL, Gatto GJ, & Stryer L (2015) *Biochemistry* (W.H. Freeman & Company, a Macmillan Education Imprint, New York) Eighth edition Ed p 1 volume (various pagings).

[2] Verkman AS (2002) Solute and macromolecule diffusion in cellular aqueous compartments. *Trends Biochem Sci* 27(1):27–33. 10.1016/s0968-0004(01)02003-5 11796221

[3] Maxfield FR & Mondal M (2006) Sterol and lipid trafficking in mammalian cells. *Biochem Soc Trans* 34(Pt 3):335–339. 10.1042/BST0340335 16709155

[4] Kholodenko BN (2003) Four-dimensional organization of protein kinase signaling cascades: the roles of diffusion, endocytosis and molecular motors. *Journal of Experimental Biology* 206(12):2073–2082. 12756289 10.1242/jeb.00298

[5] Pesaresi P, Schneider A, Kleine T, & Leister D (2007) Interorganellar communication. *Current Opinion in Plant Biology* 10(6):600–606. 10.1016/j.pbi.2007.07.007 17719262

[6] Lodish HF (2000) *Molecular cell biology* (W.H. Freeman, New York) 4th Ed pp xxxvi, 1084, G-1017, I-1036 p.

[7] Stam S & Gardel ML (2014) Cutting through the noise: the mechanics of intracellular transport. *Dev Cell* 30(4):365–366. 10.1016/j.devcel.2014.08.013 25158851

[8] Shimmen T & Yokota E (2004) Cytoplasmic streaming in plants. *Curr Opin Cell Biol* 16(1):68–72. 10.1016/j.ceb.2003.11.009 15037307

[9] Keren K, Yam PT, Kinkhabwala A, Mogilner A, & Theriot JA (2009) Intracellular fluid flow in rapidly moving cells. *Nat Cell Biol* 11(10):1219–1224. 10.1038/ncb1965 19767741 PMC2867054

[10] Goldstein RE & van de Meent JW (2015) A physical perspective on cytoplasmic streaming. *Interface Focus* 5(4):20150030 10.1098/rsfs.2015.0030 26464789 PMC4590424

[11] Sherwin RP, Richters A, & Richters V (1967) The occurrence of a cyclosis-like phenomenon in human lung cancer cells in vitro. *Cancer Res* 27(1):152–158. 5335873

[12] Parte S, et al (2014) Dynamics associated with spontaneous differentiation of ovarian stem cells in vitro. *J Ovarian Res* 7:25 10.1186/1757-2215-7-25 24568237 PMC4234975

[13] Yoshiyama S, Ishigami M, Nakamura A, & Kohama K (2009) Calcium wave for cytoplasmic streaming of Physarum polycephalum. *Cell Biol Int* 34(1):35–40. 10.1042/CBI20090158 19947949

[14] Pieuchot L, et al (2015) Cellular Subcompartments through Cytoplasmic Streaming. *Dev Cell* 34(4):410–420. 10.1016/j.devcel.2015.07.017 26305593

[15] Kamiya N & Kuroda K (1956) Velocity Distribution of the Protoplasmic Streaming in Nitella Cells. *Shokubutsugaku Zasshi* 69(822):544–554.

[16] Goldstein RE, Tuval I, & van de Meent JW (2008) Microfluidics of cytoplasmic streaming and its implications for intracellular transport. *Proc Natl Acad Sci U S A* 105(10):3663–3667. 10.1073/pnas.0707223105 18310326 PMC2268784

[17] Jitts HM CD; Stephens K; Strickland JDH. (1964) The cell division rates of some marine phytoplankters as a function of light and temperature. *J*. *Fish Res Board Can*. 21(1):139–157.

[18] Martinez J, Georgoff I, Martinez J, & Levine AJ (1991) Cellular localization and cell cycle regulation by a temperature-sensitive p53 protein. *Genes Dev* 5(2):151–159. 10.1101/gad.5.2.151 1995413

[19] Seymour RS (2001) Biophysics and physiology of temperature regulation in thermogenic flowers. *Biosci Rep* 21(2):223–236. 10.1023/a:1013608627084 11725871

[20] Patel D & Franklin KA (2014) Temperature-regulation of plant architecture. *Plant Signaling & Behavior* 4(7):577–579. 10.4161/psb.4.7.8849 PMC2710546 19820338

[21] Kiessling TR, Stange R, Kas JA, & Fritsch AW (2013) Thermorheology of living cells-impact of temperature variations on cell mechanics. *New Journal of Physics* 15.

[22] Quinn PJ (1988) Effects of temperature on cell membranes. *Symp Soc Exp Biol* 42:237–258. 3077859

[23] Pietruszka M & Lewicka S (2007) Effect of temperature on plant elongation and cell wall extensibility. *Gen Physiol Biophys* 26(1):40–47. 17579253

[24] McCabe KM & Hernandez M (2010) Molecular thermometry. *Pediatr Res* 67(5):469–475. 10.1203/PDR.0b013e3181d68cef 20139796 PMC2892932

[25] Okabe K, Sakaguchi R, Shi B, & Kiyonaka S (2018) Intracellular thermometry with fluorescent sensors for thermal biology. *Pflugers Arch* 470(5):717–731. 10.1007/s00424-018-2113-4 29397424 PMC5942359

[26] Okabe K, et al (2012) Intracellular temperature mapping with a fluorescent polymeric thermometer and fluorescence lifetime imaging microscopy. *Nat Commun* 3:705 10.1038/ncomms1714 22426226 PMC3293419

[27] Uchiyama S, et al (2015) A cationic fluorescent polymeric thermometer for the ratiometric sensing of intracellular temperature. *Analyst* 140(13):4498–4506. 10.1039/c5an00420a 25988198

[28] Hayashi T, Fukuda N, Uchiyama S, & Inada N (2015) A cell-permeable fluorescent polymeric thermometer for intracellular temperature mapping in mammalian cell lines. *PLoS One* 10(2):e0117677 10.1371/journal.pone.0117677 25692871 PMC4333297

[29] Tanimoto R, et al (2016) Detection of Temperature Difference in Neuronal Cells. *Sci Rep* 6:22071 10.1038/srep22071 26925874 PMC4772094

[30] Chretien D, et al (2018) Mitochondria are physiologically maintained at close to 50 degrees C. *PLoS Biol* 16(1):e2003992 10.1371/journal.pbio.2003992 29370167 PMC5784887

[31] Lamond AI & Earnshaw WC (1998) Structure and function in the nucleus. *Science* 280(5363):547–553. 10.1126/science.280.5363.547 9554838

[32] Smiley ST, et al (1991) Intracellular heterogeneity in mitochondrial membrane potentials revealed by a J-aggregate-forming lipophilic cation JC-1. *Proc Natl Acad Sci U S A* 88(9):3671–3675. 10.1073/pnas.88.9.3671 2023917 PMC51514

[33] Andrews ZB, Diano S, & Horvath TL (2005) Mitochondrial uncoupling proteins in the CNS: in support of function and survival. *Nat Rev Neurosci* 6(11):829–840. 10.1038/nrn1767 16224498

[34] Doxsey S (2001) Re-evaluating centrosome function. *Nat Rev Mol Cell Biol* 2(9):688–698. 10.1038/35089575 11533726

[35] Andersen JS, et al (2003) Proteomic characterization of the human centrosome by protein correlation profiling. *Nature* 426(6966):570–574. 10.1038/nature02166 14654843

[36] Bal NC, et al (2012) Sarcolipin is a newly identified regulator of muscle-based thermogenesis in mammals. *Nat Med* 18(10):1575–1579. 10.1038/nm.2897 22961106 PMC3676351

[37] Lorenz EN & World Meterological Organization. (1967) *The nature and theory of the general circulation of the atmosphere* (World Meteorological Organization, Geneva) pp xxvi, 161 p.

[38] Reid JL (2005) On the world-wide circulation of the deep water from the North Atlantic Ocean. *J Mar Res* 63(1):187–201.

[39] Fultz D (1959) *Studies of thermal convection in a rotating cylinder with some implications for large-scale atmospheric motions* (American Meteorological Society, Boston,) p 104p.

[40] Todd P (1989) Gravity-Dependent Phenomena at the Scale of the Single Cell. *Gravitational and Space Biology* 2(1). 11540086

[41] Huber MD & Gerace L (2007) The size-wise nucleus: nuclear volume control in eukaryotes. *J Cell Biol* 179(4):583–584. 10.1083/jcb.200710156 17998404 PMC2080922

[42] Geuzaine C & Remacle JF (2009) Gmsh: A 3-D finite element mesh generator with built-in pre- and post-processing facilities. *Int J Numer Meth Eng* 79(11):1309–1331.

[43] Temam R (1977) *Navier-Stokes equations*: *theory and numerical analysis* (North-Holland Pub. Co.; sole distributors for the U.S.A. and Canada, Elsevier North-Holland, Amsterdam; New York, New York) pp x, 500 p.

[44] Dhatt G, Touzot G, & Lefranç E (2012) *Finite element method* (ISTE; Wiley, London, Hoboken, N.J.) pp x, 600 p.

[45] Patankar SV (1980) *Numerical heat transfer and fluid flow* (Hemisphere Pub. Corp.; McGraw-Hill, Washington, New York) pp xiii, 197 p.

[46] Hughes TJR, Scovazzi G, & Tezduyar TE (2010) Stabilized Methods for Compressible Flows. *J Sci Comput* 43(3):343–368.

[47] Luby-Phelps K (2000) Cytoarchitecture and physical properties of cytoplasm: volume, viscosity, diffusion, intracellular surface area. *Int Rev Cytol* 192:189–221. 10.1016/s0074-7696(08)60527-6 10553280

[48] Luby-Phelps K, et al (1993) A novel fluorescence ratiometric method confirms the low solvent viscosity of the cytoplasm. *Biophys J* 65(1):236–242. 10.1016/S0006-3495(93)81075-0 8369435 PMC1225719

[49] Milo R, Jorgensen P, Moran U, Weber G, & Springer M (2010) BioNumbers—the database of key numbers in molecular and cell biology. *Nucleic Acids Res* 38(Database issue):D750–753. 10.1093/nar/gkp889 19854939 PMC2808940

[50] Crank J., "The Mathematics of Diffusion" (Oxford University Press, 1956; 2nd ed 1976)

[51] Lampo TJ, Stylianidou S, Backlund MP, Wiggins PA, & Spakowitz AJ (2017) Cytoplasmic RNA-Protein Particles Exhibit Non-Gaussian Subdiffusive Behavior. *Biophys J* 112(3):532–542. 10.1016/j.bpj.2016.11.3208 28088300 PMC5300785

[52] Brangwynne CP, Koenderink GH, MacKintosh FC, & Weitz DA (2008) Cytoplasmic diffusion: molecular motors mix it up. *J Cell Biol* 183(4):583–587. 10.1083/jcb.200806149 19001127 PMC2582900

[53] TheEngineeringToolbox (2017) Water—Thermodynamic Properties. (http://www.engineeringtoolbox.com/water-thermal-properties-d_162.html).

[54] Nakano M, et al (2017) Genetically encoded ratiometric fluorescent thermometer with wide range and rapid response. *PLoS One* 12(2):e0172344 10.1371/journal.pone.0172344 28212432 PMC5315395

[55] Feric M & Brangwynne CP (2013) A nuclear F-actin scaffold stabilizes ribonucleoprotein droplets against gravity in large cells. *Nature Cell Biology* 15(10):1253–U1295. 10.1038/ncb2830 23995731 PMC3789854

[56] Kowalewski TN P; Pierini F; Zembrzycki K; Pawlowska S. (2016) Micro and nano fluid mechanics *Advances in Mechanics: Theoretical*, *Computational and Interdisciplinary Issues*, ed al Ke (Taylor & Franics Group, London).

[57] Bird RB (1960) *Transport phenomena* (Wiley, New York,) p 780 p.

[58] Gallegos AD & Malaga C (2017) Natural convection in eccentric spherical annuli. *Eur J Mech B-Fluid* 65:464–471.

[59] Scurtu N FB, Egbers C. (2008) Three-dimensional natural convection in spherical annuli. *Journal of Physics*: *Conference Series* 137.

[60] Baffou G, Rigneault H, Marguet D, & Jullien L (2014) A critique of methods for temperature imaging in single cells. *Nat Methods* 11(9):899–901. 10.1038/nmeth.3073 25166869

[61] Al-Mehdi AB, et al (2012) Perinuclear mitochondrial clustering creates an oxidant-rich nuclear domain required for hypoxia-induced transcription. *Sci Signal* 5(231):ra47 10.1126/scisignal.2002712 22763339 PMC3565837

[62] Berdanier CD (2005) *Mitochondria in health and disease* (Taylor & Francis/CRC Press, Boca Raton, FL) pp xix, 619 p.

