# Supplementary figures and images for: Cytoplasmic convection currents and intracellular temperature gradients
Source: PLoS Comput Biol. 2019 Nov 4;15(11):e1007372. doi: 10.1371/journal.pcbi.1007372 (PMC6827888; doi:10.1371/journal.pcbi.1007372)

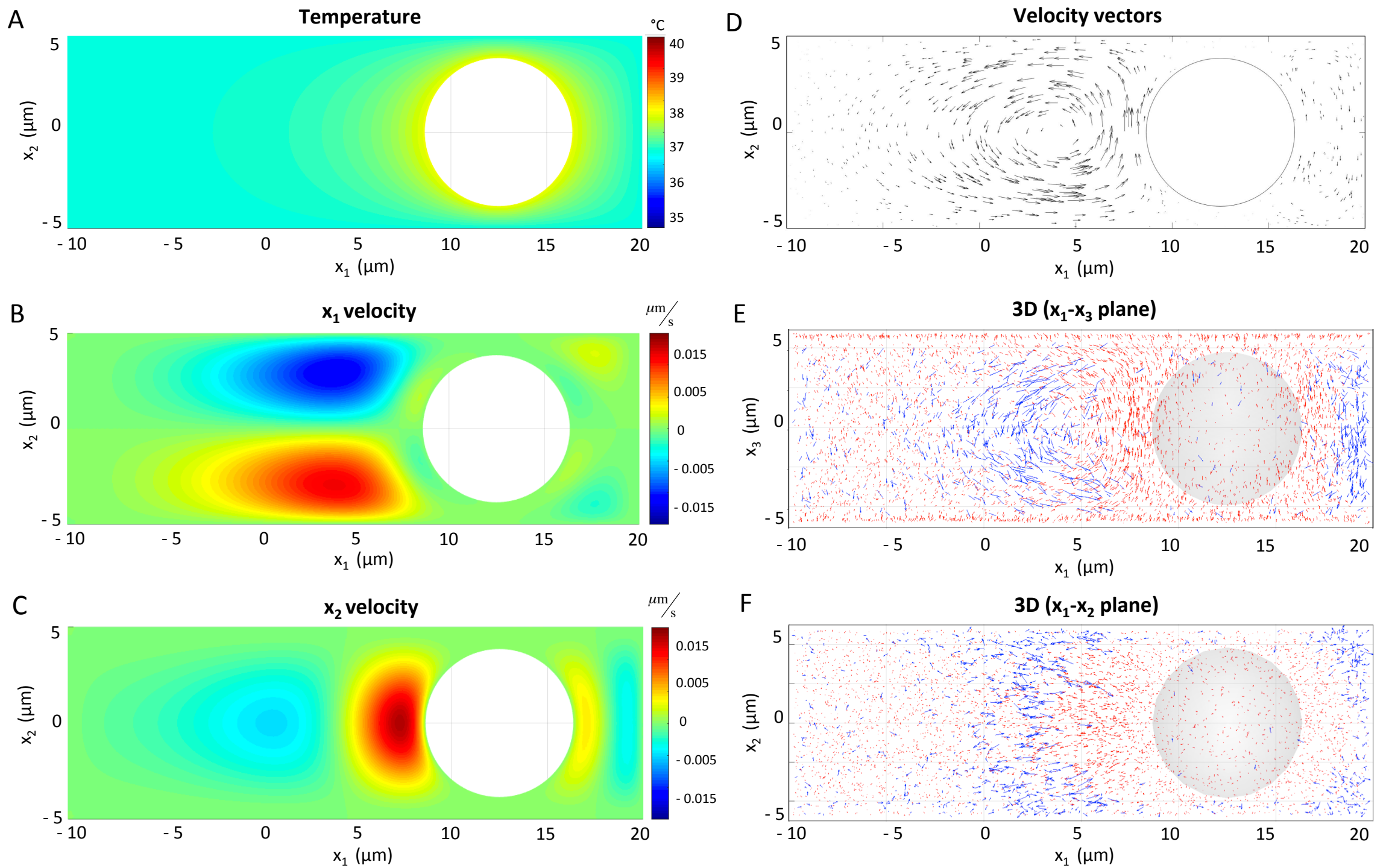

Supplement: S1 Fig — A. Temperature profile across two-dimensional domain, with heated nuclear wall (38°C) and neutral cell membrane (37°C). B,C. Two-dimensional convection-induced horizontal (B; x1) and vertical (C; x2) velocity profiles, again demonstrating one major (left of nucleus) and one minor (right of nucleus) convective circulation structure characterized by central upwelling (red) and fountain-like downwelling (blue) around the outer cell wall. D. 2D velocity vectors with arrow length indicative of speed relative to maximum speed in domain. E,F. Equivalent velocity vectors on both vertical (E) and horizontal (F) planes in three-dimensional simulations. Again, vectors with a positive vertical (x3) component are shown in red, while those with a negative vertical component are shown in blue. (PDF) [file pcbi.1007372.s001.pdf]

# Concentration

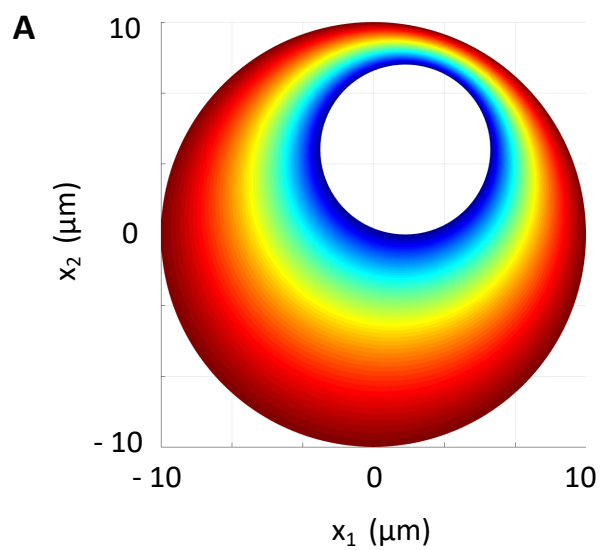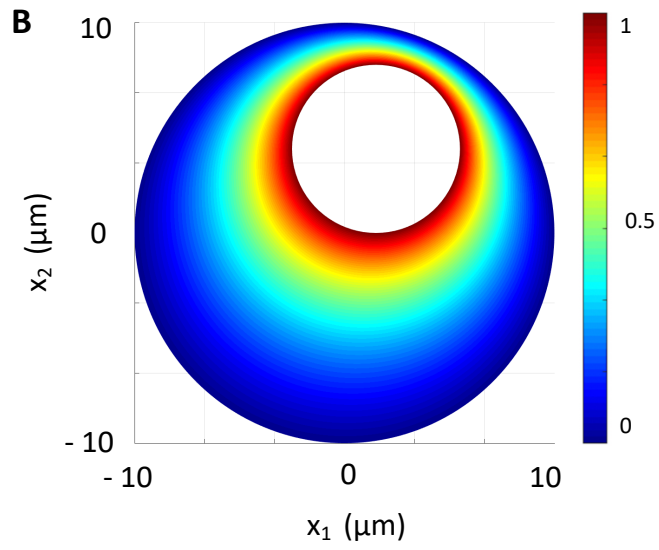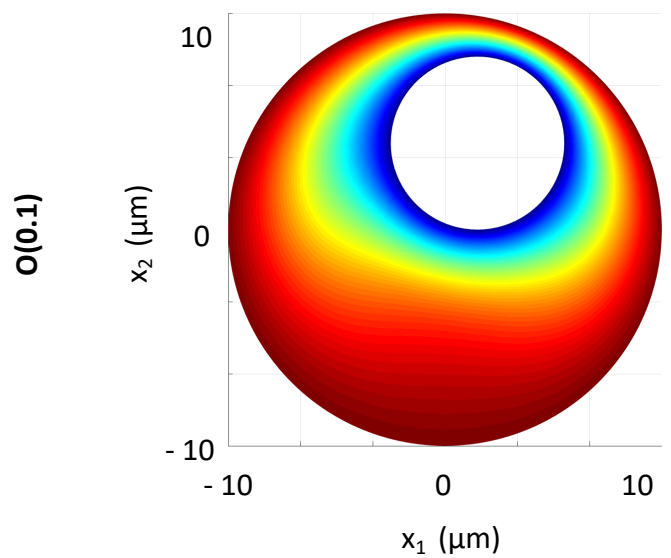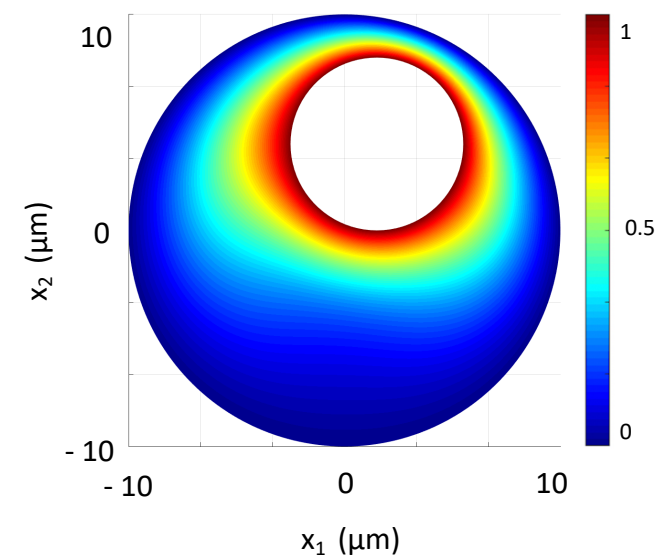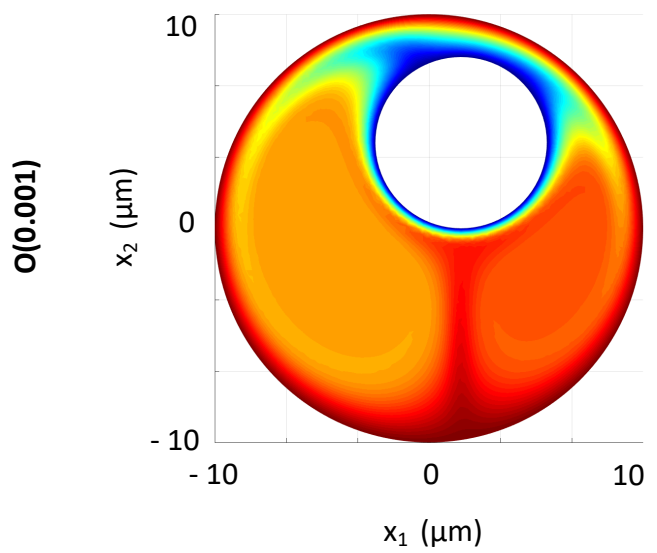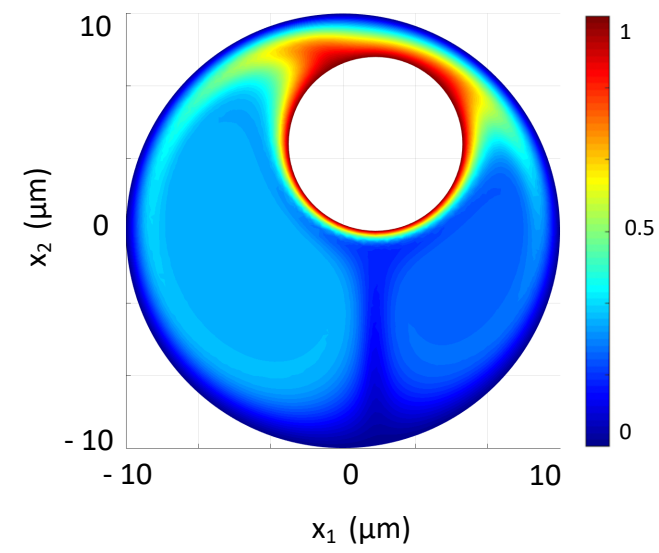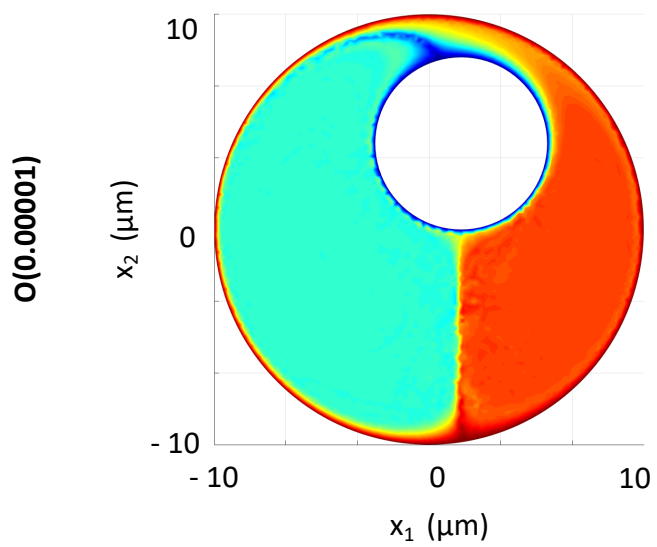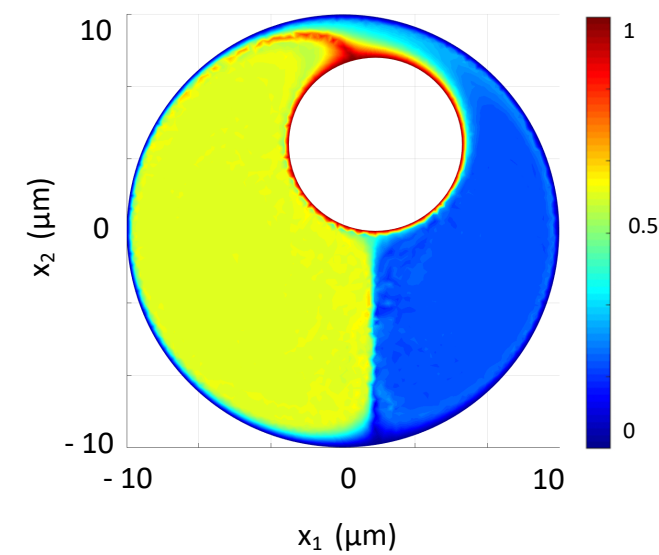

Diffusion Coefficient

Supplement: S2 Fig — In column A, the flow of this arbitrary substance is from outside the cell (high concentration imposed at outer cell wall), and in column B, the substance is originating from the nucleus (high concentration imposed at nuclear wall). At a diffusivity of 0.1 μm2/s, the concentration field begins to be influenced by convection. At a diffusivity of 0.001 μm2/s, the concentration field is clearly aligned with the flow velocity profile. At low diffusivities (0.00001 μm2/s, it can be seen that material originating at the cell membrane, and material originating at the nucleus, tend to cluster on opposite sides of the cell as a result of the offset nucleus and uneven distribution of the “convective cell” structure. (PDF) [file pcbi.1007372.s002.pdf]

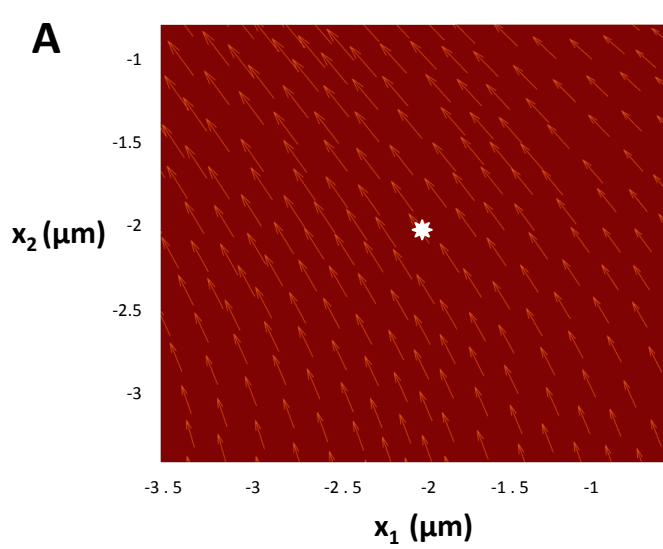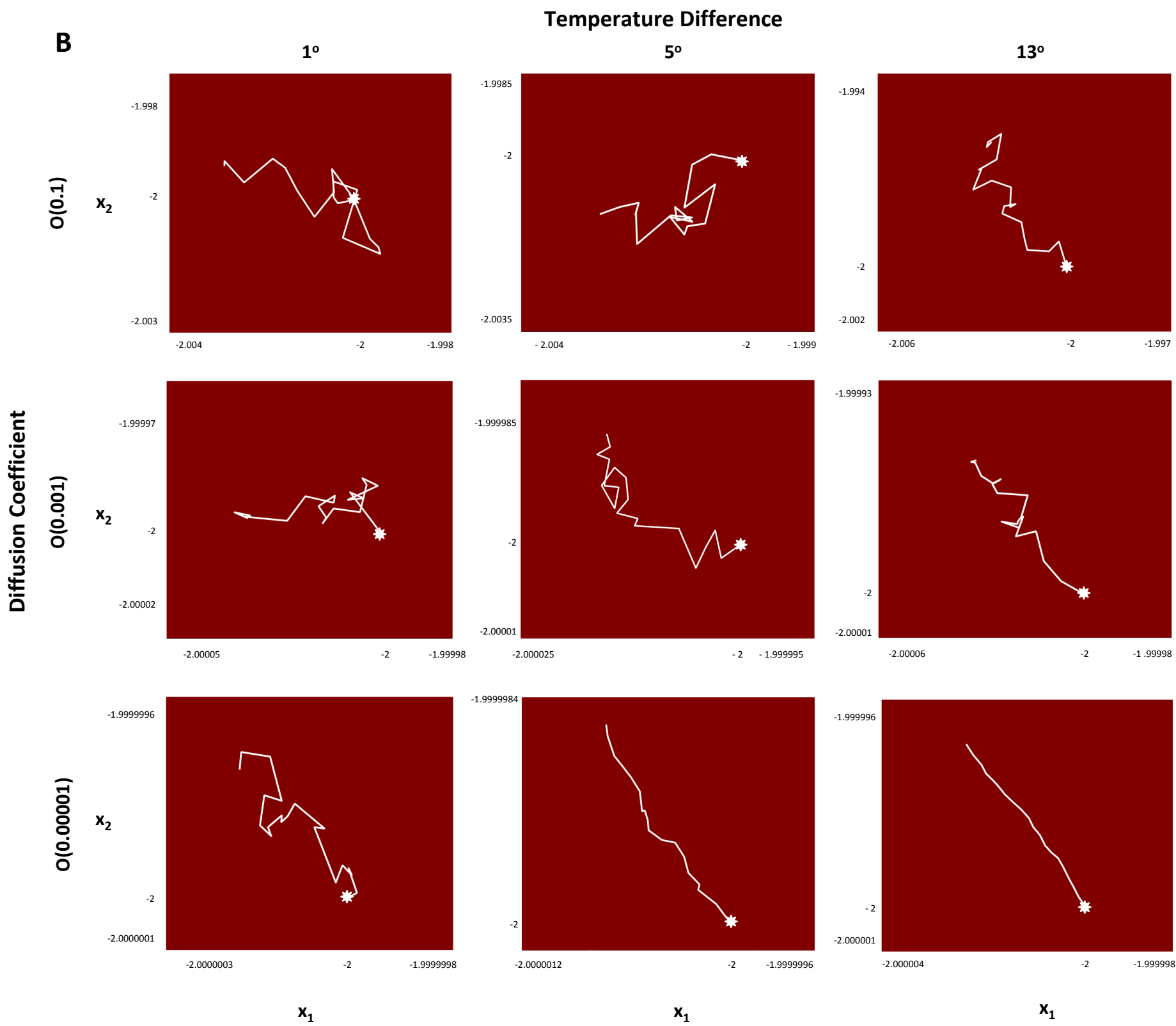

Supplement: S3 Fig — A. Zoomed-in view of start point for all simulated particle trajectories ([-2,-2], indicated by white star). Red arrows indicate that the bulk convection-driven flow is in the direction of the upper left corner of the panel. B. Individual particle trajectories (from start point again indicated by white star) for a particle of density 1050 kg/m3 and diameter 0.1 μm in flows governed by diffusion coefficients ranging from order 0.1 to order 0.00001 and temperature gradients ranging from 1°C to 13°C. Diffusion coefficients < O(0.001) or temperature gradients > 3°C are required for convection-dominated flow with this specific set of parameter values. (PDF) [file pcbi.1007372.s003.pdf]
